# The association of blood urea nitrogen-to-creatinine ratio and in-hospital mortality in acute ischemic stroke patients with atrial fibrillation: data from the MIMIC-IV database

**DOI:** 10.3389/fneur.2024.1331626

**Published:** 2024-08-07

**Authors:** Bowen Li, Juan Li, Xin Meng, Shu Yang, Furong Tian, Xiang Song, Junjie Liu

**Affiliations:** ^1^College of Clinical Medicine, North China University of Science and Technology, Tangshan, China; ^2^College of Basic Medical Sciences, North China University of Science and Technology, Tangshan, China

**Keywords:** atrial fibrillation, acute ischemic stroke, MIMIC-IV database, in-hospital mortality, intensive care unit

## Abstract

**Objective:**

This research aimed to investigate the association between the blood urea nitrogen-to-creatinine (BUN/Cr) ratio and the rate of in-hospital mortality in patients with acute ischemic stroke (AIS) and atrial fibrillation (AF), who are also receiving care in intensive care unit (ICU).

**Methods:**

A retrospective study was conducted using the MIMIC-IV database. We collected data on BUN/Cr levels at admission for patients with AIS and concurrent AF. To assess the association between BUN/Cr and in-hospital mortality rate, statistical analysis was conducted employing multivariable logistic regression models and restricted cubic spline models. These models were utilized to investigate the potential relationship and provide insights into the impact of BUN/Cr on the likelihood of in-hospital mortality. Interaction and subgroup analyses were performed to evaluate the consistency of the correlation.

**Results:**

There were a total of 856 patients (age ≥ 18 years) with a median age of 78.0 years, of which 466 (54.4%) were female. Out of 856 patients, 182 (21.26%) died in the hospital. Upon controlling for confounding factors, the multivariable logistic regression analysis elucidated that patients falling within the third trisection (Q3 > 22.41 mg/dL) exhibited a noticeably increased susceptibility to in-hospital mortality when contrasted with their counterparts positioned in the second trisection (Q2: 17.2–22.41 mg/dL) (OR = 2.02, 95% CI: 1.26–3.26, *p* = 0.004). A non-linear J-shaped relationship was observed between BUN/Cr at ICU admission and in-hospital mortality rate (*p* = 0.027), with a turning point at 19.63 mg/dL. In the threshold analysis, there was a 4% rise in in-hospital mortality for each 1 mg/dL increase in BUN/Cr (OR: 1.04, 95% CI: 1.01–1.06, *p* = 0.012).

**Conclusion:**

In patients with AIS complicated by AF, BUN/Cr at admission shows a J-shaped correlation with in-hospital mortality rate. When BUN/Cr exceeds 19.63 mg/dL, the in-hospital mortality rate increases.

## 1 Introduction

According to the findings of the Global Burden of Disease Study 2017, strokes rank as the second most prominent contributor to both mortality and disability worldwide (1). Among stroke cases, acute ischemic stroke stands out as the predominant form. AIS refers to a condition wherein the cerebral blood vessel experiences a sudden blockage, resulting in inadequate or disrupted blood supply to brain tissues. The impact of stroke is substantial, affecting patients, their families, and society at large. It is characterized by significant morbidity, a high prevalence of disability, elevated mortality rates, an increased risk of recurrence, and a considerable economic burden (2). The American Heart Association reports that the incidence of stroke will continue to increase by nearly 20% within 15 years, with a 30-day all-cause mortality rate as high as 10.5% (3). In China, studies have shown that approximately one-third of AIS patients develop death or disability within 3 months or 1 year (4).

Atrial fibrillation represents the prevailing type of arrhythmia (5) and stands as a significant contributor to various conditions, including stroke, heart failure, dementia, ischemic heart disease, and sudden death (6). Among these, stroke emerges as the most frequent and severe complication associated with AF. Individuals diagnosed with atrial fibrillation face a 4- to 5-fold greater risk of stroke compared to those without this condition (7). Furthermore, strokes resulting from atrial fibrillation exhibit higher rates of disability and mortality compared to non-atrial fibrillation-related strokes, and its in-hospital mortality rate is significantly higher than that of non-atrial fibrillation-related stroke patients (10.8% vs. 7.5%; unmatched population, 11.9% vs. 4.6%) (8).

Blood urea nitrogen and creatinine are biomarkers commonly utilized in clinical practice to assess renal function as they serve as end products of nitrogenous metabolism within the human body (9). Previous research findings have suggested that BUN/Cr can serve as an independent prognostic indicator for a negative patient outcome in cases of acute and chronic kidney injury (10), ischemic stroke (11), acute heart failure, and chronic heart failure (12, 13). As of late, evidence has emerged indicating that changes in the BUN/Cr ratio could independently predict outcomes in patients diagnosed with AIS during hospitalization (14). Nonetheless, there is a dearth of comprehensive investigations exploring the correlation between BUN/Cr and in-hospital mortality among individuals afflicted with both AIS and AF.

Therefore, in such a context, the primary objective of the current study was to evaluate the correlation between the BUN/Cr ratio and the rate of mortality during hospitalization within ICU for patients presenting with a combination of AIS and AF.

## 2 Materials and methods

### 2.1 Data source

This study is characterized as a retrospective cohort study utilizing data sourced from the Medical Information Mart for Intensive Care (MIMIC-IV 2.2) (15). The MIMIC-IV dataset, being a repository of clinical data derived from a single medical center, specifically the Beth Israel Deaconess Medical Center (BIDMC), encompasses extensive information regarding patients admitted to the ICU within the period spanning from 2008 to 2019. It is important to note that adherence to patient privacy regulations was strictly observed during the identification and utilization of this database, in accordance with the security measures outlined in the Health Insurance Portability and Accountability Act (HIPAA). One of the authors, Bowen Li (ID: 11800689), gained access to perform data extraction from the database upon successful completion of the Collaborative Institutional Training Initiative (CITI) examination. To prioritize patient privacy, all data were effectively anonymized, eliminating the necessity for informed consent. This study rigorously followed the guidelines set forth in the Statement for Strengthening the Reporting of Observational Studies in Epidemiology (STROBE), demonstrating a commitment to ensure thorough and transparent reporting of the study’s findings (16).

### 2.2 Study population

The code used for the data extraction process can be accessed on GitHub (17). For our study, eligibility was determined by selecting patients who experienced their initial ICU admission and were diagnosed with both AIS and AF. The criteria for diagnosing AIS and AF were based on the International Classification of Diseases, Ninth and Tenth Revisions (ICD-9 and ICD-10). Specifically, the diagnosis of acute ischemic stroke was defined by the ICD-9 codes 34,660, 34,661, 34,662, 34,663, 43,301, 43,311, 43,321, 43,331, 43,381, 43,391, 43,401, 43,411, and 43,491, as well as the ICD-10 code I63. Atrial fibrillation is defined by ICD-9 code: 42731 and ICD-10 code: I48.

**Figure 1:**
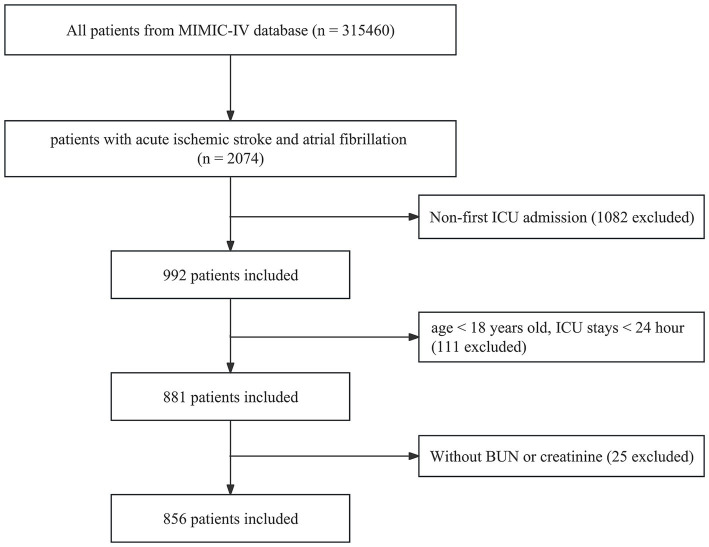
Flow chart of this study.

The inclusion criteria were as follows: (1) age ≥ 18 years old; (2) patients who were admitted to the ICU and had a duration of hospitalization exceeding 24 h; and (3) first ICU admission. The exclusion criteria for this study were as follows: (1) patients who had missing data for blood urea nitrogen and creatinine. Following the application of these criteria, the study included a total of 856 patients, as illustrated in Figure 1.

### 2.3 Data acquisition

This study utilized the PostgreSQL database management system in conjunction with Structured Query Language (SQL) for data extraction: general characteristics, vital signs, laboratory data, comorbidities, scoring system, treatment, and length of stay (LOS hospital and LOS ICU). The study outcome of interest was in-hospital mortality. Participants were tracked from the day of ICU admission to either the day of in-hospital death or discharge, whichever occurred first.

### 2.4 Statistical analysis

In this study, 856 participants were evenly divided into three groups based on baseline calculated BUN/Cr, to make the numbers roughly equal. Continuous data, such as age and laboratory parameters, were presented as either the mean ± standard deviation or median (interquartile range) depending on their distribution. Categorical data, such as gender and comorbidities, were presented as a single number (percentage). To evaluate the disparities in baseline characteristics between different groups, appropriate statistical tests were utilized. The analysis employed the ANOVA or rank-sum test for continuous variables, depending on their distribution, and the chi-squared test or Fisher’s exact test for categorical variables. These statistical tests were chosen based on the nature of the data under examination and aimed to determine whether there were statistically significant variations in baseline characteristics among the groups.

The majority of the variables under investigation display a skewed distribution, and the missing values of all variables are less than 10%. The use of the median value to fill in missing data is an effective method for handling variables that are skewed. Accordingly, median replacement was employed to address this concern. To evaluate the association between BUN/Cr ratio and in-hospital mortality, we utilized both univariate and multivariate logistic regression approaches. We investigated the impact of BUN/Cr by considering it as both a categorical variable with three levels and a continuous variable. Our regression analysis encompassed four distinct models, providing a comprehensive assessment of the relationship under scrutiny. The multifactorial models were adjusted as follows: Model 1 was not adjusted; model 2 was adjusted for age and gender; model 3 was adjusted for model 2 plus LOS hospital, DBP, MBP, temperature, Spo2, WBC, platelets, anion gap, and international normalized ratio (INR); model 4 was adjusted for model 3 plus chronic pulmonary disease, malignant cancer, severe liver disease, Oxford acute severity of illness score (OASIS), Charlson Comorbidity Index, peripheral vascular disease, cerebral edema, tracheal intubation, thrombolysis, statins, anti-platelet agents, and anticoagulant drugs.

We also used a restricted cubic spline (RCS); this approach allowed us to flexibly explore potential non-linear relationships between BUN/Cr levels and in-hospital mortality. The reference value for interpreting the results of the RCS analysis was determined as the median of the predictor variables. Upon identification of a non-linear connection, we utilized a two-segment logistic regression model to evaluate the threshold impact of BUN/Cr on in-hospital mortality.

Furthermore, we used subgroup analyses to evaluate different groups of patients. These subgroups included age, gender, peripheral vascular disease, chronic pulmonary disease, malignant cancer, severe liver disease, cerebral edema, tracheal intubation, thrombolysis, statins, anti-platelet agents, and anticoagulant drugs.

In our research, a two-tailed test was performed, and a statistical significance was defined as a *p*-value less than 0.05. All the analyses were performed with the statistical software packages R (http://www.R-project.org, The R Foundation) and Free Statistics software version 1.8 (18).

## 3 Results

### 3.1 Baseline characteristics

The study included 2074 patients in the ICU who suffered from AIS combined with AF. After applying the predetermined inclusion and exclusion criteria, 1,218 individuals were deemed ineligible and thus excluded from participating in this study. Among the excluded individuals, 1,082 individuals had previously been admitted to the ICU before their current admission, 111 individuals were under the age of 18 and had an ICU stay duration of less than 24 h, and 25 individuals did not have available data regarding their blood urea nitrogen or creatinine levels. In total, a cohort of 856 patients were included for the final data analysis. The detailed process is illustrated in Figure 1, providing a visual representation of the steps involved.

There were a total of 856 patients (age ≥ 18 years) with a median age of 78.0 years, of which 466 (54.4%) were female. Out of 856 patients, 182 (21.26%) died in the hospital. The BUN/Cr ratio was categorized into tertials based on the distribution of BUN/Cr among the patients, with 285, 285, and 286 cases in Q1 (<17.2), Q2 (17.2–22.41), and Q3 (>22.41). All the variables are listed in Table 1. Based on the analysis, we found significant differences in age, gender, heart rate, SBP, anion gap, bicarbonate, BUN, creatinine, glucose, sodium, congestive heart failure, renal disease, malignant cancer, acute physiology score III (APS III), simplified acute physiology score II (SAPS II), OASIS, and Charlson Comorbidity Index (*p* < 0.05). Patients with high levels of BUN/Cr ratio share common characteristics such as higher age, heart rate, BUN, glucose levels, APS III, SAPS II, and OASIS scores, as well as a history of malignant cancer and congestive heart failure. On the other hand, a history of renal disease shows an inverse relationship to the elevated BUN/Cr ratio levels (*p* < 0.05).

**Table 1:** Baseline characteristics related to in-hospital mortality.

Variables			Tertials of BUN/Cr			*p*-value
		Total	Q1	Q2	Q3	
			<17.2	17.2–22.41	>22.41	
Number, *n*		856	285	285	286	
Characteristics	Anchor age, (years), Median (IQR)	78.0 (69.0, 85.0)	76.0 (64.0, 83.0)	78.0 (70.0, 84.0)	80.5 (72.2, 88.0)	< 0.001
	Gender, *n* (%)					< 0.001
	Female	466 (54.4)	120 (42.1)	151 (53)	195 (68.2)	
	Male	390 (45.6)	165 (57.9)	134 (47)	91 (31.8)	
	Heart rate, (beats/min), Median (IQR)	103.0 (88.0, 122.0)	101.0 (88.0, 121.0)	99.0 (86.0, 118.0)	109.0 (90.0, 126.0)	0.005
	Temperature, (°C), Median (IQR)	37.3 (37.0, 37.7)	37.2 (37.0, 37.6)	37.3 (37.0, 37.7)	37.3 (36.9, 37.7)	0.511
	SPO_2_, (%), Median (IQR)	93.0 (91.0, 95.0)	93.0 (91.0, 95.0)	93.0 (91.0, 95.0)	92.0 (91.0, 94.8)	0.147
	SBP, (mmHg), Median (IQR)	160.0 (145.0, 177.0)	159.0 (144.0, 178.0)	164.0 (148.0, 182.0)	155.5 (144.0, 172.0)	0.002
	DBP, (mmHg), Median (IQR)	96.0 (80.0, 110.0)	97.0 (82.0, 110.0)	95.0 (79.0, 108.0)	96.0 (81.0, 111.0)	0.67
	MBP, (mmHg), Median (IQR)	111.0 (99.0, 126.0)	112.0 (98.0, 125.0)	111.0 (100.0, 126.0)	110.0 (98.0, 126.0)	0.522
	Respiratory rate, (time/min), Median (IQR)	27.0 (24.0, 31.0)	27.0 (24.0, 31.0)	27.0 (24.0, 31.0)	28.0 (24.0, 32.0)	0.067
Laboratory	Hemoglobin, (g/L), Median (IQR)	11.2 (9.4, 12.8)	11.3 (9.5, 13.1)	11.3 (9.4, 12.6)	11.0 (9.2, 12.6)	0.156
	Platelets, (10^9^/L), Median (IQR)	186.0 (141.0, 238.0)	186.0 (136.0, 235.0)	185.0 (143.0, 234.0)	187.5 (138.0, 241.0)	0.943
	Anion gap, Median (IQR)	16.0 (14.0, 18.0)	16.0 (13.0, 19.0)	15.0 (13.0, 18.0)	16.0 (14.0, 19.0)	0.02
	WBC, (10^9^/L), Median (IQR)	11.6 (8.8, 15.4)	11.2 (8.5, 14.8)	11.7 (9.2, 15.4)	12.1 (9.1, 15.8)	0.101
	Bicarbonate, (mEq/l), Median (IQR)	22.0 (20.0, 24.0)	22.0 (19.0, 24.0)	22.0 (20.0, 24.0)	22.0 (19.0, 25.0)	0.014
	BUN, (mg/dl), Median (IQR)	22.0 (16.0, 31.0)	16.0 (12.0, 23.0)	21.0 (17.0, 28.0)	27.5 (22.0, 38.0)	< 0.001
	Calcium, (mg/dl), Median (IQR)	8.4 (8.0, 8.8)	8.4 (7.9, 8.8)	8.4 (8.0, 8.9)	8.4 (8.0, 8.8)	0.383
	Chloride, (mmol/L), Median (IQR)	103.0 (100.0, 106.0)	103.0 (100.0, 106.0)	103.0 (100.0, 106.0)	103.0 (99.0, 106.0)	0.811
	Creatinine, (mg/dl), Median (IQR)	1.0 (0.8, 1.4)	1.1 (0.8, 1.6)	1.1 (0.8, 1.4)	1.0 (0.8, 1.3)	0.018
	Glucose, (mg/dl), Median (IQR)	137.0 (112.8, 179.0)	128.0 (106.0, 172.0)	137.0 (114.0, 172.0)	146.0 (121.2, 190.8)	< 0.001
	Sodium, (mEq/l), Median (IQR)	141.0 (138.0, 143.0)	140.0 (138.0, 142.0)	141.0 (138.0, 143.0)	141.0 (138.0, 144.0)	0.037
	Potassium, (mEq/l), Median (IQR)	3.9 (3.6, 4.3)	4.0 (3.6, 4.3)	3.9 (3.5, 4.2)	3.9 (3.6, 4.3)	0.275
	INR, Median (IQR)	1.3 (1.1, 1.5)	1.3 (1.1, 1.5)	1.3 (1.1, 1.5)	1.3 (1.1, 1.5)	0.476
Comorbidities	Myocardial infarct, *n* (%)	150 (17.5)	43 (15.1)	58 (20.4)	49 (17.1)	0.25
	Congestive heart failure, *n* (%)	319 (37.3)	92 (32.3)	104 (36.5)	123 (43)	0.028
	Peripheral vascular disease, *n* (%)	123 (14.4)	41 (14.4)	45 (15.8)	37 (12.9)	0.624
	Dementia, *n* (%)	54 (6.3)	11 (3.9)	23 (8.1)	20 (7)	0.1
	Chronic pulmonary disease, *n* (%)	169 (19.7)	47 (16.5)	65 (22.8)	57 (19.9)	0.166
	Rheumatic disease, *n* (%)	20 (2.3)	6 (2.1)	7 (2.5)	7 (2.4)	0.951
	Peptic ulcer disease, *n* (%)	18 (2.1)	5 (1.8)	6 (2.1)	7 (2.4)	0.847
	Mild liver disease, *n* (%)	33 (3.9)	12 (4.2)	9 (3.2)	12 (4.2)	0.756
	Diabetes, *n* (%)	272 (31.8)	91 (31.9)	86 (30.2)	95 (33.2)	0.736
	Paraplegia, *n* (%)	463 (54.1)	146 (51.2)	150 (52.6)	167 (58.4)	0.191
	Renal disease, *n* (%)	179 (20.9)	69 (24.2)	67 (23.5)	43 (15)	0.011
	Malignant cancer, *n* (%)	69 (8.1)	13 (4.6)	26 (9.1)	30 (10.5)	0.024
	Severe liver disease, *n* (%)	9 (1.1)	2 (0.7)	4 (1.4)	3 (1)	0.79
	Metastatic solid tumor, *n* (%)	29 (3.4)	5 (1.8)	11 (3.9)	13 (4.5)	0.158
	Sepsis, *n* (%)	400 (46.7)	123 (43.2)	139 (48.8)	138 (48.3)	0.332
	Cerebral edema, *n* (%)	127 (14.8)	44 (15.4)	38 (13.3)	45 (15.7)	0.679
Scoring systems	APS III, Median (IQR)	46.0 (34.0, 64.0)	41.0 (30.0, 60.0)	47.0 (34.0, 64.0)	50.0 (39.0, 66.8)	< 0.001
	SAPS II, Median (IQR)	37.0 (31.0, 46.0)	35.0 (28.0, 43.0)	37.0 (31.0, 45.0)	40.0 (33.0, 48.0)	< 0.001
	OASIS, Median (IQR)	35.0 (29.0, 42.0)	34.0 (28.0, 41.0)	35.0 (29.0, 41.0)	36.5 (31.0, 43.0)	0.013
	CCI, Median (IQR)	8.0 (6.0, 9.0)	7.0 (6.0, 9.0)	8.0 (7.0, 9.0)	8.0 (7.0, 9.0)	< 0.001
Treatment	Tracheal intubation, *n* (%)	109 (12.7)	30 (10.5)	36 (12.6)	43 (15)	0.27
	Thrombolysis, *n* (%)	74 (8.6)	17 (6)	29 (10.2)	28 (9.8)	0.141
	Statins, *n* (%)	206 (24.1)	66 (23.2)	80 (28.1)	60 (21)	0.127
	Anti-platelet agents, *n* (%)	604 (70.6)	190 (66.7)	213 (74.7)	201 (70.3)	0.106
	Anticoagulant drugs, *n* (%)	734 (85.7)	242 (84.9)	247 (86.7)	245 (85.7)	0.835
Length of stay	LOS hospital, Median (IQR)	9.7 (5.2, 17.1)	8.7 (5.0, 15.6)	9.9 (5.1, 17.0)	10.4 (5.9, 18.1)	0.109
	LOS ICU, Median (IQR)	3.6 (1.9, 7.2)	3.6 (2.0, 7.1)	3.8 (1.9, 7.2)	3.5 (2.0, 7.1)	0.773

Data were expressed as mean ± SD/median (interquartile ranges (IQR)) for continuous variables and percentage for categorical variables. SD, standard deviation; IQR, interquartile range; SPO2, saturation of percutaneous oxygen; SBP, systolic blood pressure; DBP, diastolic blood pressure; MBP, mean blood pressure; WBC, white blood cell; BUN, blood urea nitrogen; INR: international normalized ratio; APS III, acute physiology score III; SAPS II, simplified acute physiology score II; OASIS, Oxford acute severity of illness score; CCI, Charlson Comorbidity Index. LOS hospital, length of stay in the hospital; LOS ICU, length of stay in the ICU.

### 3.2 Univariate analysis

As depicted in Table 2, the univariate logistic regression analysis disclosed a positive correlation between in-hospital mortality and variables such as age, heart rate, temperature, respiratory rate, glucose level, sodium level, anion gap, WBC, renal disease, malignant cancer, metastatic solid tumor, APS III score, SAPS II score, OASIS score and Charlson Comorbidity Index, cerebral edema, and tracheal intubation. Conversely, variables including DBP, hemoglobin level, bicarbonate level, calcium level, LOS hospital, statins, and anticoagulant drugs exhibited an inverse association with in-hospital mortality.

**Table 2:** Univariate logistic analysis between BUN/Cr and in-hospital mortality.

Variable	OR (95% CI)	*p*-value
BUN/Cr	1.03 (1.02 ~ 1.05)	<0.001
Age	1.02 (1 ~ 1.03)	0.032
Gender (male)	1.03 (0.74 ~ 1.43)	0.856
Heart rate	1.02 (1.01 ~ 1.02)	<0.001
DBP	0.98 (0.97 ~ 0.99)	0.022
MBP	1 (0.99 ~ 1.01)	0.792
Respiratory rate	1.03 (1.01 ~ 1.05)	0.042
Temperature	1.61(1.26 ~ 2.05)	<0.001
SPO2	1 (0.98 ~ 1.02)	0.894
WBC	1.07 (1.04 ~ 1.1)	<0.001
Hemoglobin	0.92 (0.86 ~ 0.99)	0.019
Anion gap	1.11 (1.07 ~ 1.15)	<0.001
Bicarbonate	0.93 (0.89 ~ 0.97)	<0.001
BUN	1.02 (1.01 ~ 1.03)	<0.001
Calcium	0.8 (0.67 ~ 0.97)	0.022
Chloride	0.98 (0.96 ~ 1.01)	0.289
Creatinine	1.18 (1.05 ~ 1.32)	0.004
Glucose	1.01 (1.01 ~ 1.03)	0.003
Sodium	1.04 (1.01 ~ 1.09)	0.025
Potassium	0.9 (0.66 ~ 1.22)	0.493
INR	1.05 (0.9 ~ 1.22)	0.559
Myocardial infarct	1.44 (0.96 ~ 2.17)	0.076
Congestive heart failure	1.27 (0.91 ~ 1.78)	0.158
Peripheral vascular disease	1.51 (0.98 ~ 2.33)	0.063
Dementia	0.83 (0.41 ~ 1.69)	0.611
Chronic pulmonary disease	1.35 (0.91 ~ 1.99)	0.139
Rheumatic disease	0.92 (0.31 ~ 2.8)	0.889
Peptic ulcer disease	0.74 (0.21 ~ 2.57)	0.631
Mild liver disease	1.41 (0.64 ~ 3.09)	0.392
Diabetes	0.94 (0.66 ~ 1.34)	0.742
Paraplegia	0.77 (0.55 ~ 1.07)	0.114
Renal disease	1.48 (1.01 ~ 2.17)	0.042
Malignant cancer	1.97 (1.16 ~ 3.35)	0.012
Severe liver disease	3.01 (0.8 ~ 11.31)	0.103
Metastatic solid tumor	2.34 (1.09 ~ 5.06)	0.03
APS III	1.03 (1.03 ~ 1.04)	<0.001
SAPS II	1.06 (1.04 ~ 1.07)	<0.001
OASIS	1.1 (1.08 ~ 1.12)	<0.001
Charlson Comorbidity Index	1.12 (1.05 ~ 1.2)	0.001
LOS hospital	0.98 (0.96 ~ 1)	0.012
LOS ICU	1.01 (0.99 ~ 1.03)	0.351
Cerebral edema	2.7 (1.8 ~ 4.04)	<0.001
Tracheal intubation	3.13 (2.05 ~ 4.78)	<0.001
Thrombolysis	0.94 (0.52 ~ 1.69)	0.827
Statins	0.64 (0.42 ~ 0.97)	0.036
Anti-platelet agents	0.56 (0.4 ~ 0.78)	0.001
Anticoagulant drugs	0.76 (0.49 ~ 1.19)	0.228

DBP, diastolic blood pressure; MBP, mean blood pressure; SPO2, saturation of percutaneous oxygen; WBC, white blood cell; BUN, blood urea nitrogen; INR: international normalized ratio; APS III, acute physiology score III; SAPS II, simplified acute physiology score II; OASIS, Oxford acute severity of illness score; LOS hospital, length of stay in the hospital; LOS ICU, length of stay in the ICU.

### 3.3 Effects of the BUN/Cr ratio and in-hospital mortality

Table 3 presents both unadjusted and adjusted models for the analysis. The multivariate logistic regression model, with the second trisection of BUN/Cr serving as the reference group, revealed a noteworthy positive association between elevated BUN/Cr levels, treated as a continuous variable, and a heightened risk of in-hospital mortality among patients diagnosed with AIS and coexisting AF. After adjusting for confounders, in-hospital mortality could increase by 26% for each standard deviation increase in a patient’s BUN/Cr (OR: 1.26, 95% CI: 1.04–1.53, *p* = 0.019). When we used BUN/Cr as a categorical variable, we found that there was a higher risk of in-hospital mortality in Group 1(Q1 > 17.2) and Group 3 (Q3 > 22.41) compared to Group 2 (Q2: 17.2–22.41). This suggests that patients with BUN/Cr levels above 22.41 may have an increased probability of experiencing in-hospital mortality relative to those who fall within the range of 17.2–22.41. However, BUN/Cr in Group 1 (Q1 < 17.2) was not significantly associated with in-hospital mortality (*p* = 0.493).

**Table 3:** Association between BUN/Cr and in-hospital mortality in multiple regression model.

Variable	Model 1		Model 2		Model 3		Model 4	
	OR(95%CI)	*p*-value	OR(95%CI)	*p*-value	OR(95%CI)	*p*-value	OR(95%CI)	*p*-value
BUN/Cr per SD	1.32 (1.13 ~ 1.54)	<0.001	1.31 (1.12 ~ 1.53)	0.001	1.33 (1.12 ~ 1.57)	0.001	1.26 (1.04 ~ 1.53)	0.019
BUN/Cr tertials								
<17.2	1.08 (0.7 ~ 1.66)	0.74	1.1 (0.71 ~ 1.7)	0.681	1.13 (0.71 ~ 1.79)	0.604	1.2 (0.71 ~ 2.01)	0.493
17.2 ~ 22.41	1 (Reference)		1 (Reference)		1 (Reference)		1 (Reference)	
>22.41	2.02 (1.35 ~ 3.02)	0.001	2.03 (1.35 ~ 3.05)	0.001	2.18 (1.42 ~ 3.34)	<0.001	2.02 (1.26 ~ 3.26)	0.004
P for trend		0.001		0.002		0.002		0.025

BUN, blood urea nitrogen; Cr, creatinine; OR, odds ratio; CI, confidence interval. Model 1: no adjusted. Model 2: adjusted for gender and age. Model 3: adjusted for model 2 plus LOS hospital, DBP, MBP, temperature, Spo2, platelets, WBC, and INR. Model 4: adjusted for model 3 plus peripheral vascular disease, chronic pulmonary disease, malignant cancer, severe liver disease, OASIS, Charlson Comorbidity Index, cerebral edema, tracheal intubation, thrombolysis, statins, anti-platelet agents, and anticoagulant drugs.

**Figure 2:**
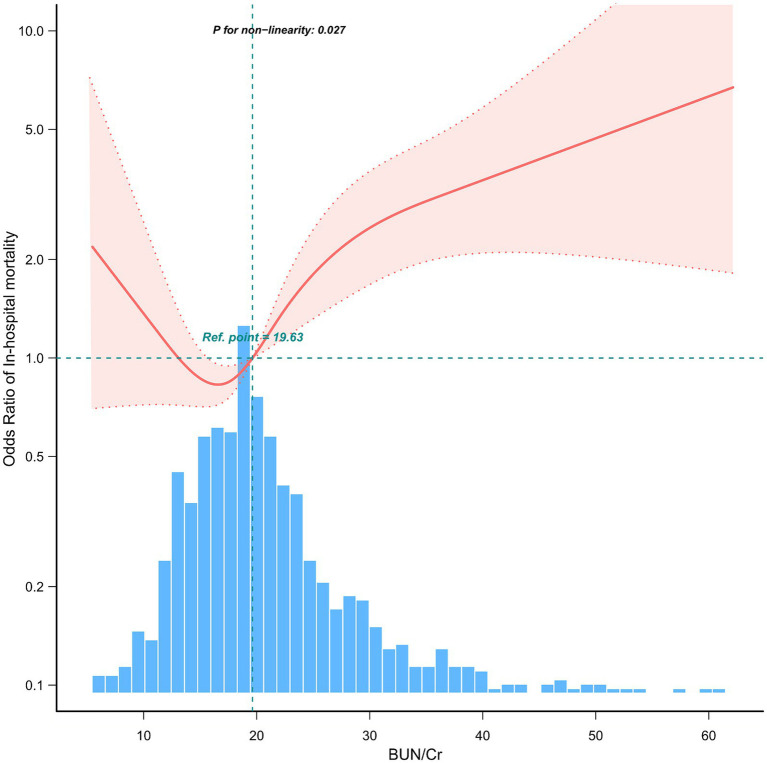
Adjusting factors included gender, age, LOS hospital, DBP, MBP, temperature, Spo2, platelets, WBC, INR, anion gap, peripheral vascular disease, chronic pulmonary disease, malignant cancer, severe liver disease, OASIS, Charlson Comorbidity Index, cerebral edema, tracheal intubation, thrombolysis, statins, anti-platelet agents, and anticoagulant drugs.

In this study, we observed a non-linear relationship between BUN/Cr and patients with AIS combined with AF, and after adjusting for gender, age, LOS hospital, DBP, MBP, temperature, SPO2, platelets, WBC, INR, chronic pulmonary disease, malignant cancer, severe liver disease, peripheral vascular disease, OASIS, Charlson Comorbidity Index, cerebral edema, tracheal intubation, thrombolysis, statins, anti-platelet agents, and anticoagulant drugs, restricted cubic spline analysis showed a J-shaped association between BUN/Cr and in-hospital mortality (Figure 2, *p* = 0.027).

Based on our two-stage logistic regression analysis, we discovered an inflection point at 19.63 mg/dL for BUN/Cr (Table 4). There was no association between BUN/Cr levels (< 19.63 mg/dL) and in-hospital mortality in patients presenting with a combination of AIS and AF (*p* = 0.127), and specifically in patients with BUN/Cr > 19.63 mg/dL, there was a 4% rise in in-hospital mortality for each 1 mg/dL increase in BUN/Cr (OR: 1.04, 95% CI: 1.01–1.06, *p* = 0.012).

**Table 4:** Threshold analysis of BUN/Cr on in-hospital mortality using a two-segment regression model.

Threshold of BUN/Cr, mg/dl	OR	95%CI	*p*-value
<19.63	0.94	0.87–1.02	0.127
≥19.63	1.04	1.01–1.06	0.012
likelihood Ratio test			0.027

BUN, blood urea nitrogen; Cr, creatinine; OR, odds ratio; CI, confidence interval. Adjusting factors included gender, age, LOS hospital, DBP, MBP, temperature, Spo2, platelets, WBC, INR, anion gap, peripheral vascular disease, chronic pulmonary disease, malignant cancer, severe liver disease, OASIS, Charlson Comorbidity Index, cerebral edema, tracheal intubation, thrombolysis, statins, anti-platelet agents, and anticoagulant drugs.

### 3.4 Subgroup analysis

**Figure 3:**
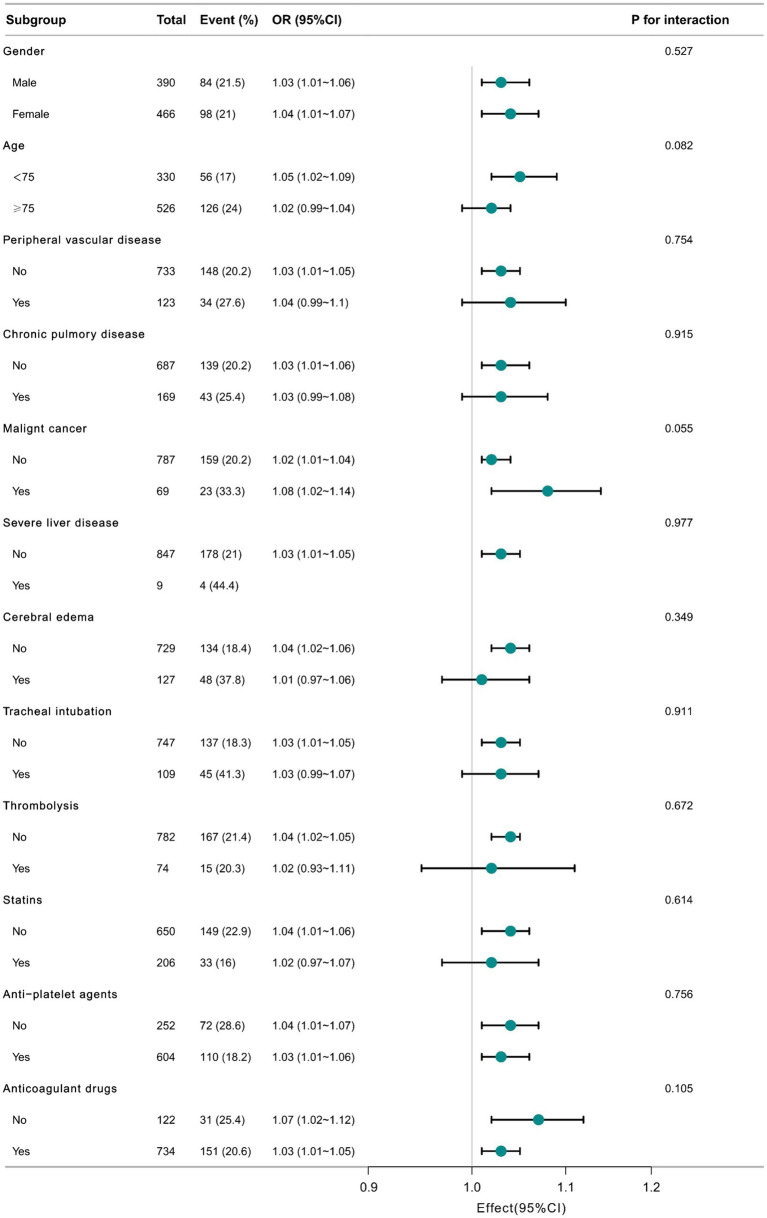
Relationship between BUN/Cr and in-hospital mortality in subgroup analysis.

As shown in Figure 3, we performed subgroup analyses to assess the relationship between BUN/Cr and in-hospital mortality. No significant interaction was observed after subgroup analyses based on confounders such as age (<75 and ≥ 75 years old), gender, peripheral vascular disease, COPD, malignant cancer, severe liver disease, cerebral edema, tracheal intubation, thrombolysis, statins, anti-platelet agents, and anticoagulant drugs (all *p*-values for interaction >0.05).

## 4 Discussion

Through multifactorial logistic regression modeling, our retrospective analysis revealed a positive correlation between BUN/Cr and in-hospital mortality among ischemic stroke patients with comorbid AF. Of particular interest, patients with higher BUN/Cr values exhibited increased risk of mortality.

The BUN/Cr ratio possesses considerable clinical utility due to its ease of acquisition and ability to serve as a marker for both neurohumoral activity and renal function. Literature preceding this study has consistently demonstrated the importance of BUN/Cr in prognosticating the likelihood of ischemic stroke occurrence. Akimoto et al. showed that the cause of the development of cerebral infarction may be due to the disproportionate increase in BUN/Cr in patients with cerebral infarction (19). Within the domain of AIS research, Deng et al. unearthed a compelling link between BUN/Cr and the occurrence of 3-month mortality, particularly evident in individuals with elevated HDL levels (20). In addition, Kunal Bhatia et al. elucidated the independent prognostic capacity of a BUN/Cr ratio surpassing 15 mg/dL in predicting neurological deterioration within the acute ischemic stroke population (21). Previous studies have examined the possibility that an increased BUN/Cr may be associated with a poorer prognosis in critically ill patients. Huang et al. reportedly found a non-linear correlation in patients with acute myocardial infarction, and according to the KM curve, the highest in-hospital mortality was observed when BUN/Cr ≥18.34 mg/dL (22). Furthermore, a research conducted by Zhu et al. performed a study involving 509 hospitalized individuals afflicted with acute heart failure. Their research elucidated that the BUN/Cr ratio could independently anticipate all-cause mortality, while a heightened BUN/Cr ratio was indicative of an adverse prognosis (23).

Recently, several studies have also identified a role for BUN/Cr as a prognostic factor in cardiovascular system disorders. Activation of the neurohormonal system, which inhibits the reabsorption of urea nitrogen, includes vasopressin, the renin-angiotensin-aldosterone system (RAAS), and the sympathetic nervous system. Thus, BUN/Cr, serving as a biomarker of neurohormonal activity, has exhibited associations with prognostic endpoints related to cardiac dysfunction (9, 24, 25). The measurement of BUN/Cr ratio has been found to provide valuable insights into the prognosis and outcomes of individuals with cardiac dysfunction. Its relationship with various prognostic endpoints highlights its potential as a useful biomarker for assessing cardiac health and predicting clinical outcomes in this context. A study by Qin et al. found that higher levels of urea nitrogen were associated with an increased incidence of atrial fibrillation in women. This finding adds to the evidence that urea nitrogen is also a risk factor for atrial fibrillation in older adults (26).

Stroke is globally recognized as the second most prevalent cause of mortality and a significant contributor to disability. Among different types of strokes, ischemic stroke stands out as the most frequent, constituting approximately 87% of all reported cases (27). Indeed, AF is widely recognized as a significant risk factor for ischemic stroke. It has been reported that more than 20% of ischemic stroke cases are attributable to cardiac thromboembolism resulting from atrial fibrillation (28). Furthermore, the prevalence of atrial fibrillation among stroke patients continues to rise annually. Individuals with atrial fibrillation may face an annual incidence rate of ischemic stroke reaching up to 18.2%, given the concurrent presence of diverse risk factors (29). It is true that strokes linked to atrial fibrillation are more likely to result in fatality or disability than strokes with other causes, and they have the highest in-hospital mortality rate among ischemic strokes (8). Moreover, prompt recognition and management of atrial fibrillation can help prevent thromboembolic events, which are strongly associated with the occurrence of stroke. Therefore, early detection and swift implementation of appropriate emergency interventions hold paramount significance for individuals affected by the combined occurrence of ischemic stroke and AF. These proactive measures are instrumental in minimizing the risks of disability and fatality.

The precise underlying mechanism linking BUN/Cr to in-hospital mortality in patients presenting with acute ischemic stroke and concomitant atrial fibrillation remains uncertain. In light of this uncertainty, we propose several potential mechanisms that may contribute to this association. BUN/Cr represents a vital metric for the evaluation of dehydration, and its significance is particularly noteworthy within the demographic of ischemic stroke patients. Given its association with a high likelihood of unfavorable outcomes upon hospital discharge, dehydration emerges as a prevalent occurrence among such individuals. Dehydration is known to decrease cerebral perfusion and disrupt neuroplasticity (30, 31), consequently exhibiting a correlation with recurrent embolic strokes and thrombotic events, including venous thromboembolism, subsequent to an acute stroke. The BUN/Cr ratio can provide insight into heightened dehydration levels and may serve as a valuable clinical marker for assessing the progression of an acute stroke. Furthermore, emerging evidence suggests a significant bidirectional correlation between the heart and the kidneys. On the one hand, diminished renal function can contribute to the development of atrial fibrillation through elevated RAAS activity, inflammation, and the promotion of cardiovascular conditions such as coronary artery disease (CHD) and heart failure. On the other hand, atrial fibrillation can be induced by RAAS activation, thromboembolism, hypoperfusion, inflammation, and the onset of other cardiovascular disorders (32). This intricate interplay underscores the complex relationship between these two vital organ systems. According to researchers, there is a suggestion that urea nitrogen could potentially serve as a marker for the “renal response” triggered by neurohormonal activation. Consequently, it may provide insights into the underlying pathophysiological processes associated with cardiovascular disease (CVD) (33). Engin Akgul et al. found that BUN and Cr levels were higher, respectively, in the postoperative AF group, suggesting that renal insufficiency (RI) paves the way for many other potentially life-threatening diseases in patients (34). At the same time, there is growing evidence indicating a potential association between BUN/Cr levels and oxidative stress as well as endothelial dysfunction in patients. These additional mechanisms could contribute to the observed relationship and shed further light on the underlying physiological processes.

Our investigation corroborates the aforementioned findings, where our current study demonstrates a curvilinear correlation between BUN/Cr and patients who exhibit acute ischemic stroke alongside comorbid atrial fibrillation. Notably, a J-shaped relationship was identified, signifying that heightened BUN/Cr values were linked to a heightened likelihood of in-hospital mortality among individuals diagnosed with AIS and AF, consistent with previous research findings on BUN/Cr. The BUN/Cr ratio is easily obtainable from clinical practice, and these observations serve to emphasize the substantial prognostic value of the BUN/Cr ratio within this particular cohort of patients, enabling timely identification of high-risk patients and the development of better treatment strategies.

### 4.1 Strengths and limitations

Our study possesses several notable strengths that contribute to advancing the current understanding in this field.

First, based on the information we have gathered so far, the present study is the first to reveal the association between BUN/Cr and in-hospital mortality in patients with AIS combined with AF. The findings derived from our investigation unveiled a non-linear relationship, specifically manifested as a J-shaped association, between BUN/Cr and in-hospital mortality. Notably, as BUN/Cr exceeded the threshold of 19.63 mg/dL, there was a gradual and progressive escalation in the risk of mortality. These findings may serve as a theoretical underpinning and provide supportive evidence for the therapeutic management of these patients.

Second, to discern the non-linear correlation between BUN/Cr and in-hospital mortality among individuals afflicted with AIS concomitant with AF, the methodology employed in this investigation encompassed the utilization of restricted cubic spline analysis.

Third, to improve statistical robustness and minimize the incidence of chance events, we used BUN/Cr as both a continuous and categorical variable. This approach improves the reliability of the final results through a more comprehensive analysis.

In addition, we undertook a threshold effect analysis to investigate the potential threshold value that may exist in the correlation between BUN/Cr and in-hospital mortality. We also conducted a subgroup analysis to examine the correlation between BUN/Cr and in-hospital mortality within specific subgroups.

Our study also has some limitations. First, the laboratory data utilized in this study were prospectively collected on the initial day of ICU admission; therefore, we were unable to analyze ongoing changes in the BUN/Cr ratio.

Second, despite controlling for certain known confounding factors, it is important to acknowledge that our study outcomes might have been influenced by other unmeasured or unidentified variables.

Third, it is essential to acknowledge that this study was conducted as a single-center retrospective analysis. Due to the limited study population to a specific country and intensive care unit, the representativeness of patients included is relatively poor, resulting in an inherent risk of selection bias that cannot be completely avoided. Due to the nature of retrospective studies, the sample size is relatively limited and there is confounding bias. However, we used multiple logistic regression analysis to correct for the effects of confounding factors, to improve the scientific validity of the research results. Nevertheless, further validation is required through multicenter prospective studies with larger sample sizes and longer time spans.

Fourth, it is important to note that while we can observe an association between BUN/Cr and in-hospital mortality, we cannot establish a direct causal relationship between the two variables. Nevertheless, there is substantial evidence indicating a statistically significant correlation between BUN/Cr and in-hospital mortality.

Fifth, due to limitations in the database itself, the study did not include specific criteria for patients to be admitted to the ICU and the most common causes of death in this group.

## 5 Conclusion

In this study, we observed that BUN/Cr within 24 h of admission to the ICU for patients with AIS combined with AF was J-shaped associated with in-hospital mortality. The risk of in-hospital mortality increases when the BUN/Cr ratio > 19.63 mg/dL. Therefore, more attention should be paid to patients with high BUN/Cr levels, who may have higher in-hospital mortality. This will benefit clinicians and contribute to better decision-making.

## Data availability statement

The data analyzed in this study was obtained from the Medical Information Mart for Intensive Care IV (MIMIC-IV) database, the following licenses/restrictions apply: to access the files, users must be credentialed users, complete the required training (CITI Data or Specimens Only Research) and sign the data use agreement for the project. Requests to access these datasets should be directed to PhysioNet, https://physionet.org/, doi: 10.13026/6mm1-ek67.

## Ethics statement

Ethical review and approval was not required for the study on human participants in accordance with the local legislation and institutional requirements. Written informed consent from the patients/participants or patients/participants legal guardian/next of kin was not required to participate in this study in accordance with the national legislation and the institutional requirements.

## Author contributions

BL: Conceptualization, Data curation, Formal analysis, Writing – original draft. JuaL: Data curation, Writing – review & editing. XM: Data curation, Writing – review & editing. SY: Data curation, Writing – review & editing. FT: Data curation, Writing – review & editing. XS: Data curation, Writing – review & editing. JunL: Supervision, Writing – review & editing.
